# What is Central Toxic Keratopathy Syndrome if it is not Diffuse lamellar Keratitis Grade IV?

**DOI:** 10.4103/0974-9233.61218

**Published:** 2010

**Authors:** Ribhi Hazin, Yassine J. Daoud, Yousuf M. Khalifa

**Affiliations:** Massachusetts Eye and Ear Infirmary, Harvard Medical School, Boston, Massachusetts, USA; 1 The Wilmer Eye Institute, Johns Hopkins University School of Medicine, Baltimore, USA; 2 University of Utah School of Medicine, Salt Lake City, UT, USA

**Keywords:** Central toxic keratopathy, diffuse lamellar keratitis, PRK, keratitis, Laser *in situ* keratomileusis

## Abstract

The Central Toxic Keratopathy (CTK) syndrome describes a rare, acute, self-limited, non-inflammatory process that yields central corneal opacification and significant hyperopic shift after refractive surgery. Despite being exceedingly rare, certain clinical features of CTK give the condition a striking resemblance to other more serious inflammatory conditions, including diffuse lamellar keratitis (DLK). As the authors demonstrate in this article, despite the overlapping clinical features, CTK is a disease process that is distinct from DLK and, therefore, in need of distinct management interventions.

## INTRODUCTION

The Central Toxic Keratopathy (CTK) syndrome describes a rare, acute, non-inflammatory process that results in dense opacification of the central corneal stroma after refractive surgery.[1–3] Refractive surgeries are widely accepted and commonly performed throughout the world;[4] fortunately, CTK and other complications remain uncommon after refractive surgery with only 0.0076-0.016% of patients exhibiting such complications postoperatively.[5,6] In addition to the characteristic central corneal opacification that arises 3-9 days after refractive surgery, CTK typically presents with stromal tissue loss[3] and significant hyperopic refractive shift.[7] Although striae are a characteristic feature of CTK,[7] the condition can exist in the absence of striae.[3] The findings in CTK typically resolve between 2-18 months.[8]

## DIFFUSE LAMELLAR KERATITIS GRADE IV OR CTK?

**Figure 1:**
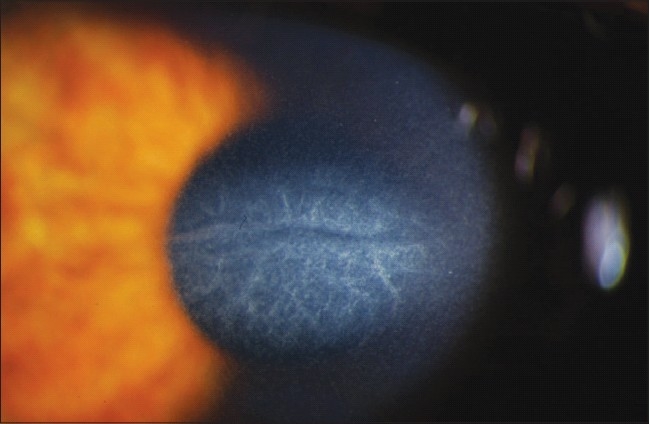
Characteristic loss of corneal clarity in CTK. Image Courtesy of Majid Moshirfar, M.D.

The characteristic loss of corneal clarity and clinical appearance in CTK bears a resemblance to a number of inflammatory and infectious conditions including contact lens-induced keratitis [Figure 1],[8] infectious keratitis,[3] post-photorefractive keratectomy (PRK) haze,[1] diffuse lamellar keratitis (DLK)[1,7] and corneal haze secondary to increased intraocular pressure.[3] In addition to corneal opacification, DLK, like CTK, can result in a hyperopic shift.[2] Furthermore, and perhaps more importantly, CTK is oftentimes preceded by DLK[9] particularly during postoperative days 1 and 2.[1,9] Given the overlapping clinical features of the two conditions, CTK has been erroneously conflated with—and often mis-diagnosed as—severe, grade IV DLK.[7] Notwithstanding, CTK and DLK can be distinguished by inciting factors,[7] timing of onset[1] and by the nature of clinical findings[3] each disease characteristically exhibits.

DLK is characterized by infiltrates in the LASIK flap interface postoperatively, yet the exact pathophysiologic mechanism of CTK remains unclear.[1,2] Some researchers have hypothesized that CTK may arise as a result of a toxic reaction catalyzed by laser photoactivation of povidone-iodine,[3] meibomian gland secretions,[3] talc from latex surgical gloves,[3] or post-surgical debris from the microkeratome blade.[8] The duration of laser exposure as well as the type of laser used in refractive procedures have also been posited as potential causes of CTK.[10] Despite the recent groundswell of emerging theories on possible etiologies of CTK, the lack of concrete evidence from controlled, clinical trials suggest that the exact molecular or biochemical basis for the findings in CTK remains uncertain.[1]

Since CTK is non-inflammatory in nature, appropriate management of the condition varies from the inflammatory and infectious conditions CTK mimics. Thus, distinguishing CTK from these other conditions remains critical to ensuring optimal outcomes in patients with suspected CTK. DLK grade IV, the condition CTK is most often confused with, can be readily differentiated from CTK via recognition of clinical features as well as through careful observation and attention to onset of clinical findings.[1] The characteristic clinical presentation in DLK develops on postoperative day 1 or 2 with mild to moderate inflammatory cells within the central or peripheral flap interface and resolving within 5-8 days.[1–3] The post-surgical haze that accompanies DLK tends to be nonlocalized[2,5] and subepithelial.[1]

Conversely, the centralized corneal haze in CTK occurs 3-9 days after refractive surgery [1,3] and is often preceded by DLK on postoperative day 1 or 2.[3,9] Unlike DLK which is confined to the flap interface, CTK extends anteriorly or posteriorly from the interface.[3] While DLK is known to resolve within 5-8 days, CTK can persist for up to 18 months.[1,3,9]

Although distinguishing CTK from DLK grade 4 can be a challenge, close monitoring of disease progression can yield important distinguishing features for each disease. For instance, DLK grade 4 becomes apparent after topical medication, multiple flap lifts, or multiple irrigations fail to suppress the interface inflammatory component.[2] More importantly, many eyes with DLK grade 4 typically develop classic clinical manifestations of DLK grade 1-2 over the first two weeks with the characteristic peripheral lesion gradually transforming into central grade 3 and grade 4 over the course of 3-5 weeks.[2,3] DLK Grade 4 rarely occurs within the first 3 post-operative weeks whereas CTK yields classic findings within the first 3-7 days after surgery.[1,9] As will be demonstrated below, these findings have important implications for the management of CTK.

Although the focus of this paper centers on elaborating the distinguishing features between CTK and DLK, a brief overview on infectious keratitis is warranted especially since CTK has been mistaken for infectious keratitis in the past.[7,9] While the corneal opacification in CTK can persist for months, the opacification progressively improves over time. Conversely, the corneal haze in infectious keratitis tends to become more opacified over time and spreads anteriorly and posteriorly to involve the corneal flap and stroma.[7,11] Further, patients with CTK tend to exhibit “quiet” eyes while those with infectious keratitis often demonstrate an active anterior chamber reaction,[9,12] purulent discharge[13] and inflammatory cells within and around the opacity.[1,13]

**Figure 2:**
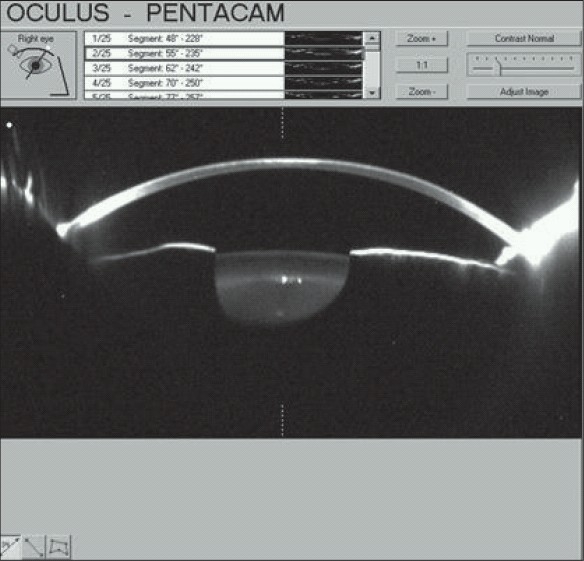
A Pentacam image revealing significant central thinning and keratometric flattening in a patient with CTK. Image Courtesy of Majid Moshirfar, M.D.

Although lifting the flap and irrigating the interface can provide benefit in reducing inflammation in early stages of DLK, given the non-inflammatory nature of CTK, irrigation of the interface does not confer benefits in CTK haze.[9] In fact, irrigating beneath the flap in CTK can be deleterious insofar as it may exacerbate existing tissue necrosis, precipitate a buttonhole of the flap, increase the chance for epithelial ingrowths, and lead to keratocycte apoptosis in the stromal bed.[1,3] Furthermore, irrigation of the flap can increase thinning of the residual stromal bed leading to an early increase in hyperopia due to augmented anterior corneal flattening [Figure 2].[1,3] The undesirable outcomes associated with raising a post-surgical flap (i.e., development of a buttonhole from tissue loss and the potential for decreased BCVA), should be given prior consideration in patients in whom the diagnosis is uncertain. Given its non-inflammatory nature the utility of aggressive therapy with topical corticosteroids has been questioned by investigators,[8] some of whom view corticosteroids as absolutely contraindicated in the treatment of CTK.[1,3]

Notwithstanding, the ambiguous etiology of CTK has led to a lack of consensus on what constitutes optimal management for the condition. Although Sonmez *et al* and Moshirfar *et al*, suggest reserving corticosteroids until a definitive diagnosis is made,[3,8] some investigators have recently suggested early, empirical topical corticosteroid therapy for all patients suspected of either DLK and CTK.[9] The rationale behind administering empirical corticosteroids in both DLK and CTK, stems from the desire to minimize irreversible complications that can arise from a failure to reduce inflammation in DLK. Put simply, if a patient had CTK and topical corticosteroids were applied for 2-3 weeks, the adverse effects of such short-term therapy is often negligible. Conversely, if a patient had DLK and was misdiagnosed as having CTK, the failure to administer topical corticosteroids early in the development of DLK could have potentially disastrous consequences on the patient's vision.

## CURRENT PERSPECTIVES

A lack of consensus exists both in terms of accurately identifying CTK as well as in best management practices for the condition. Whether or not CTK exists as a distinct entity or if it is, in fact, a part of the DLK spectrum remains unresolved. Given the overlapping clinical features of the two conditions, further research involving examination of involved eyes with confocal microscopy may be warranted in order to tease out the true nature of CTK and, thus, direct future management decisions based on objective scientific findings. At present no treatment for CTK has been validated as standard of care therapy by randomized, controlled trials.

## CONCLUSIONS

Although rare, CTK describes a self-limited, non-inflammatory process that occurs after LASIK and other refractive surgical procedures. Given the overlapping clinical features it shares with other conditions, CTK is often mistaken for more serious inflammatory and infectious disease processes. Specifically, CTK bears a striking resemblance to DLK, and may often be preceded by DLK, prompting some experts to suggest that CTK is, in fact, a variant of DLK. Still other experts point to the lack of inflammatory changes and the distinct onset of clinical findings in CTK as compelling evidence supporting the claim that CTK is a disease process that is distinct from DLK. This lack of consensus on the nature of CTK has also led to a commensurate lack of agreement on what constitutes appropriate management of the condition. The current uncertainty surrounding the management of CTK underscores the need for randomized, controlled trials to establish standard of care therapy for CTK.
